# Supplementary material for: Tunable Donor–Acceptor Linear Conjugated Polymers Involving Cyanostyrylthiophene Linkages for Visible-Light-Driven Hydrogen Production
Source: Molecules. 2023 Feb 27;28(5):2203. doi: 10.3390/molecules28052203 (PMC10004844; doi:10.3390/molecules28052203)
Supplement: Supplementary file 1 [file molecules-28-02203-s001.zip › molecules-2213393-supplementary/SI for molecules 2023. 2 11.pdf]

Supporting Information

for

Tunable Linear Donor-Acceptor Conjugated Polymers with Cyanostyrylthiophene Linkages for Visible-Light Driven Hydrogen Evolution

Dongnai Ye [1,2], Lei Liu [1], Yujie Zhang [1], Jiabin Qiu [1,*], Zhirong Tan [1], Yuqin Xing [1] and Shiyong Liu [1,*]

[1] Jiangxi Provincial Key Laboratory of Functional Molecular Materials Chemistry, College of Materials, Metallurgical and Chemistry, Jiangxi University of Science and Technology, Ganzhou 341000, China

[2] School of Chemistry and Chemical Engineering, Gannan Normal University, Ganzhou 341000, China

* Correspondence: jiabinqiu@jxust.edu.cn (J.Q.); chelsy@jxust.edu.cn or chelsy@zju.edu.cn (S.L.)

Structure	Optimized geometries	The dihedral angles
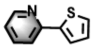	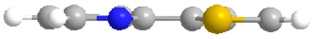	1.2°
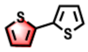	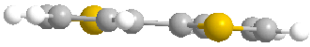	1.5°
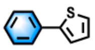	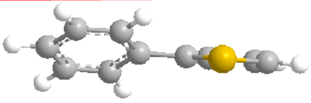	30.1°
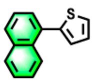	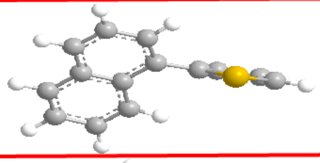	52.4°
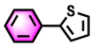	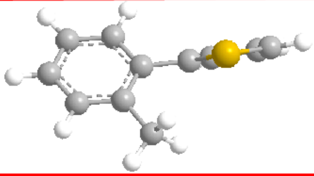	53.6°

Figure S1. Optimized geometries of the donor unit of **CP1-CP5** and thiophenyl unit by DFT calculation.

Structure	Optimized geometries	The dihedral angles
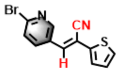 CST-BPD	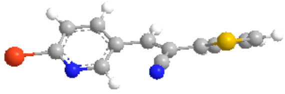	28.0°
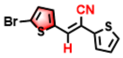 CST-BT	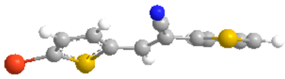	26.0°
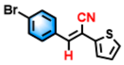 CST-BP	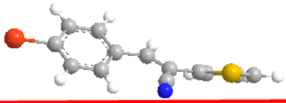	23.9°
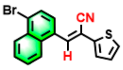 CST-BN	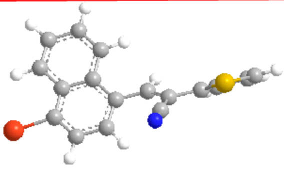	46.4°
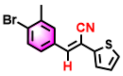 CST-BMP	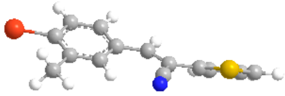	15.1°

Figure S2. Optimized geometries of monomers predicated by DFT calculation.

Structure	Optimized geometries	The dihedral angles
Dimer-CST-BPD	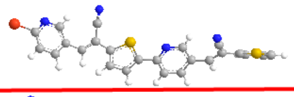	29.0°
Dimer-CST-BT	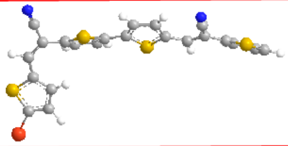	28.1°
Dimer-CST-BP	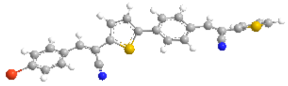	20.8°
Dimer-CST-BN	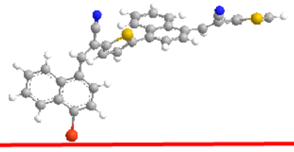	43.1°
Dimer-CST-BMP	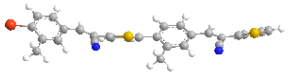	19.9°

Figure S3. Optimized geometries of dimer molecule system predicated by DFT calculation.

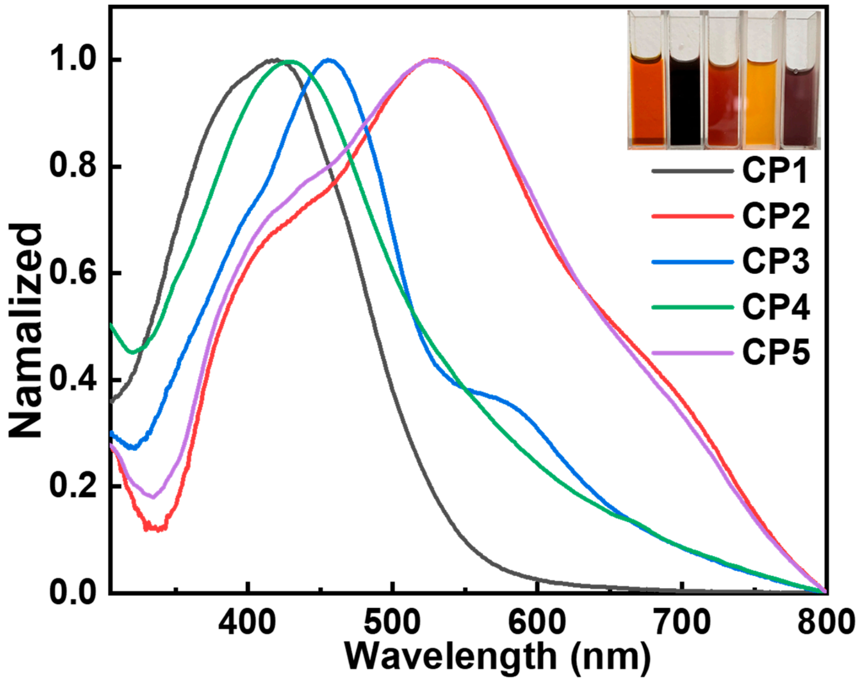

Figure S4. UV-vis spectra when CPs were dispersed in NMP and photographs of CPs were dispersed in NMP.

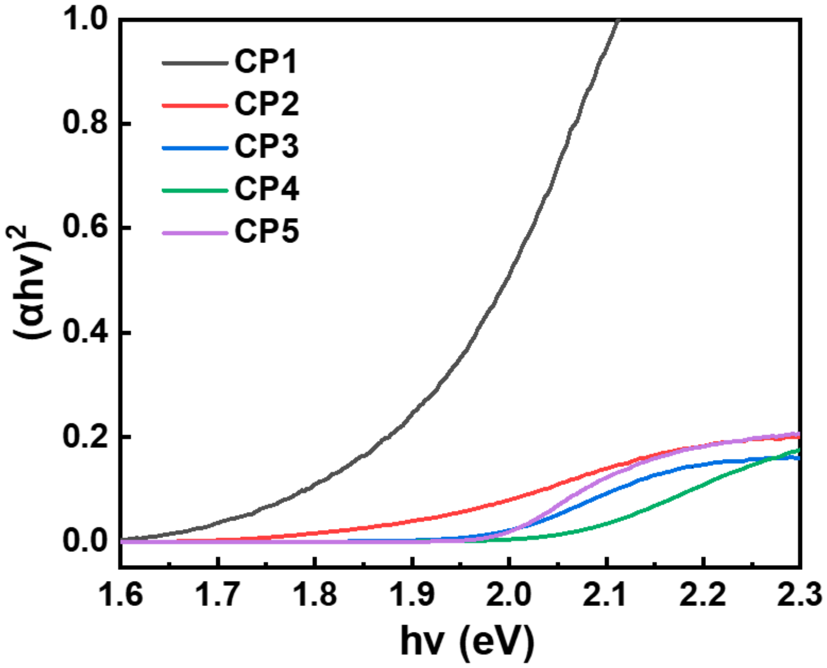

Figure S5. Tauc plots of the transformed Kubelka–Munk function vs energy of **CP1-CP5**.

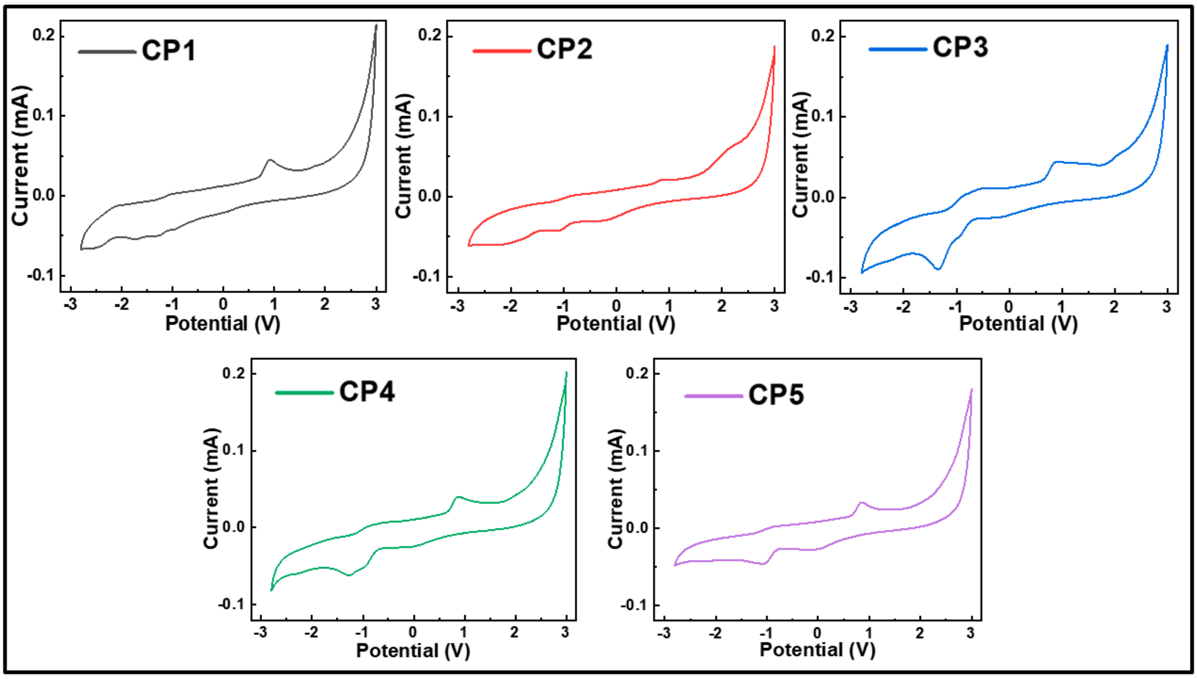

Figure S6. CV curves of the as-prepared **CP1-CP5**.

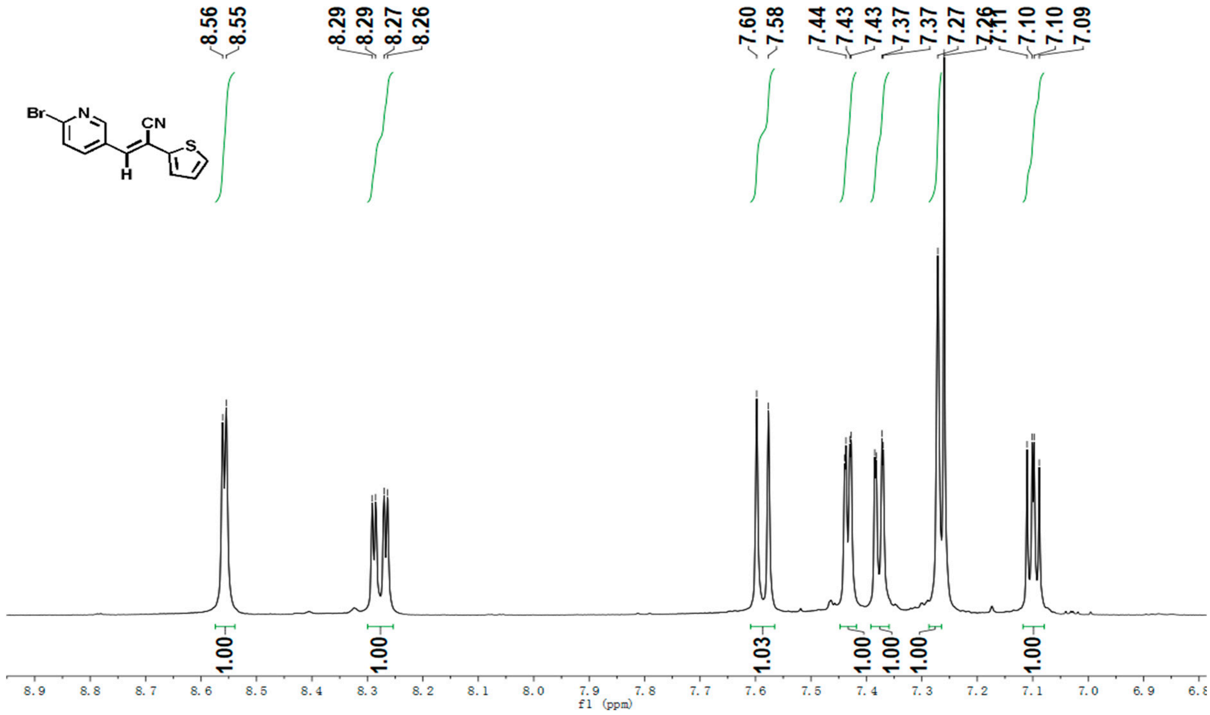

Figure S7. ^{1}H NMR spectrum of **CST-BPD** (400 MHz, $CDCl_3$).

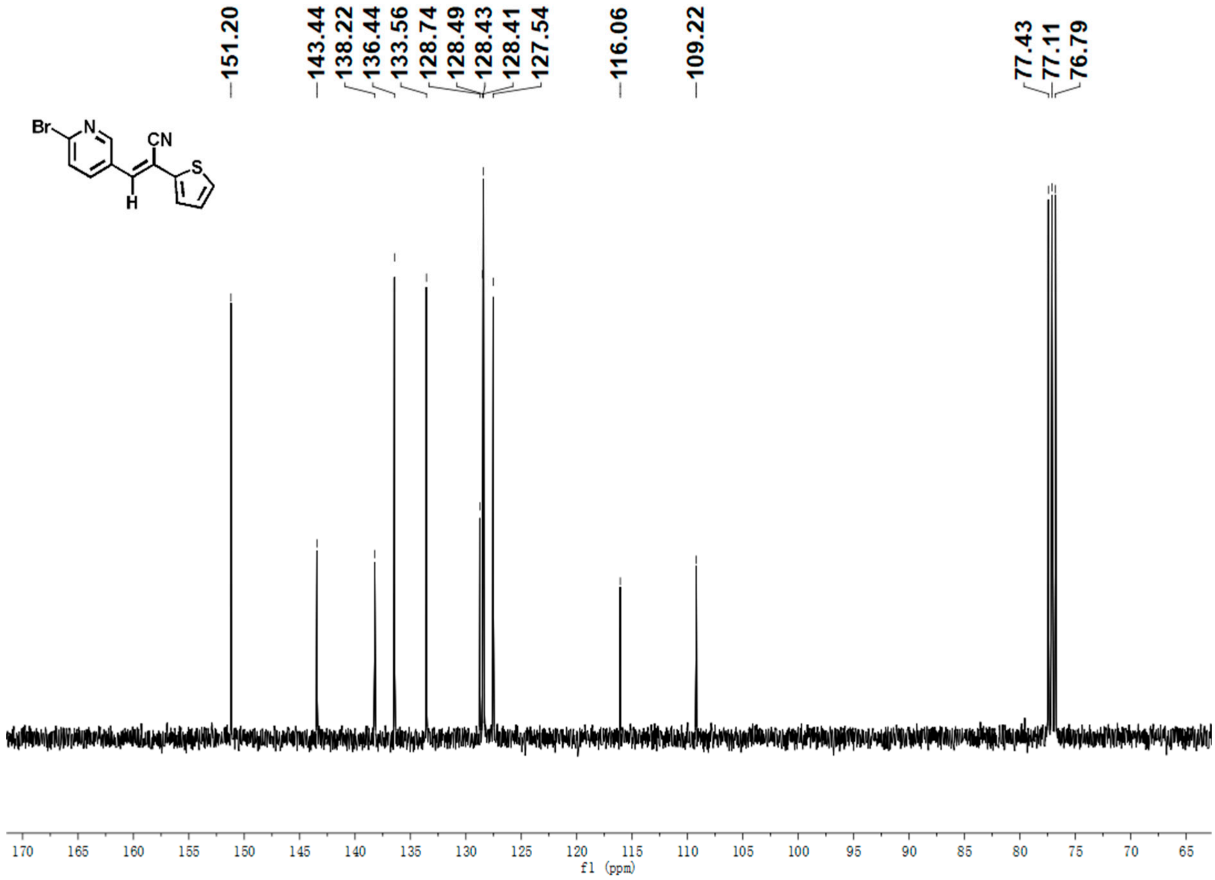

Figure S8. $^{13}C\{^{1}H$ NMR$\}$spectrum of **CST-BPD** (100 MHz, $CDCl_3$).

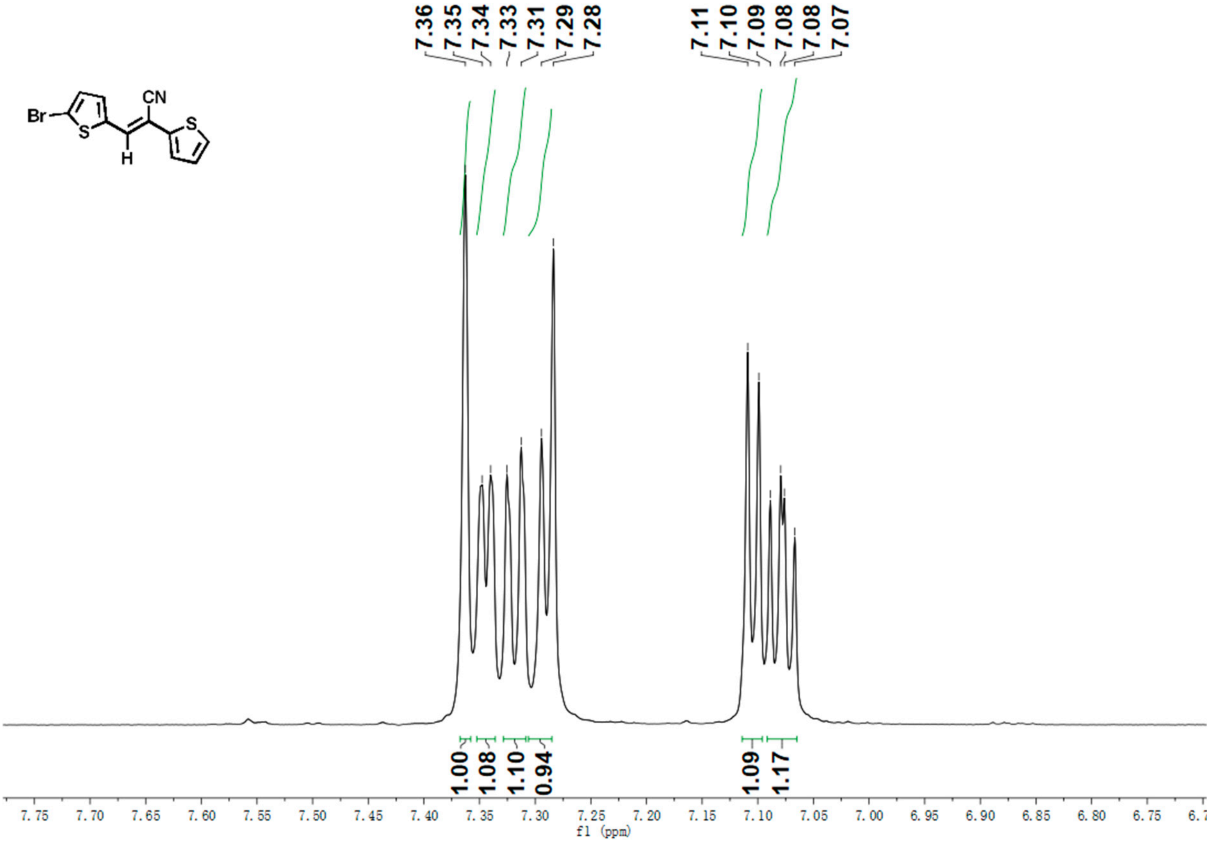

Figure S9. ^{1}H NMR spectrum of **CST-BT** (400 MHz, $CDCl_3$).

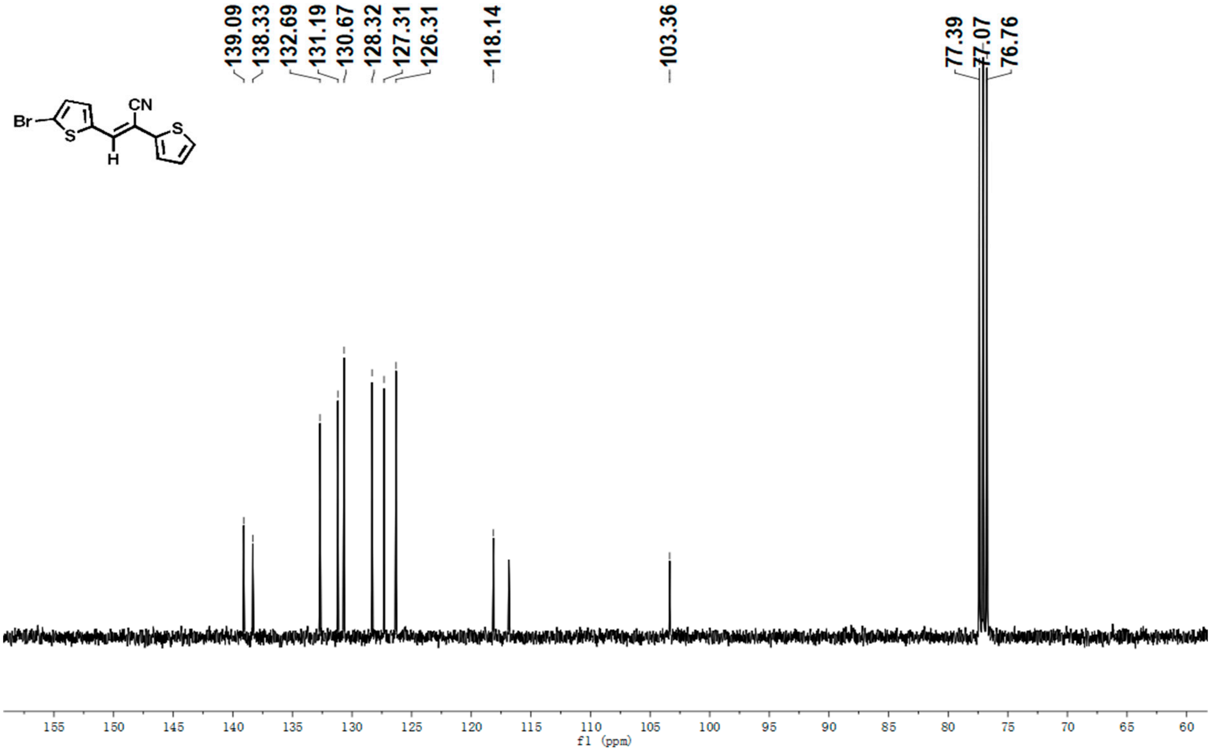

Figure S10. $^{13}C\{^{1}H$ NMR}spectrum of **CST-BT** (100 MHz, $CDCl_3$).

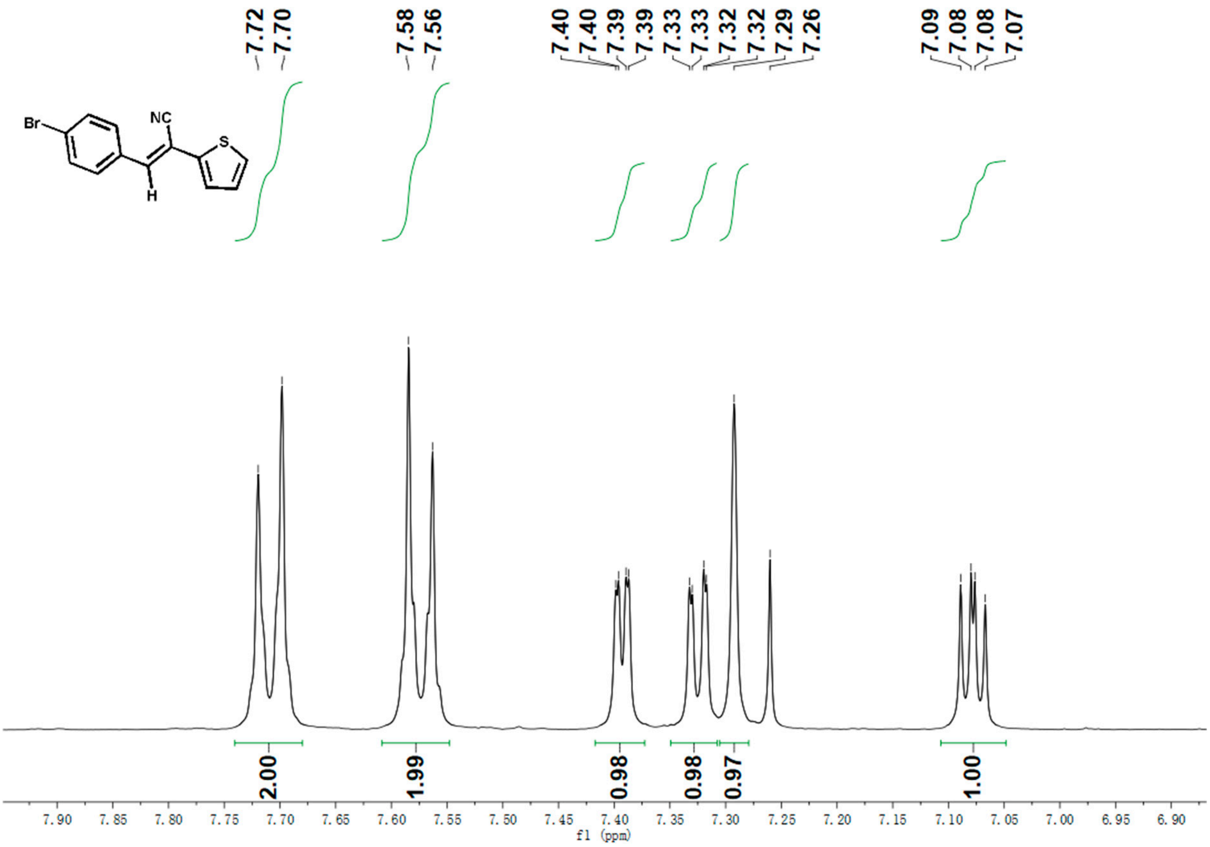

Figure S11. ^{1}H NMR spectrum of **CST-BP** (400 MHz, $CDCl_3$).

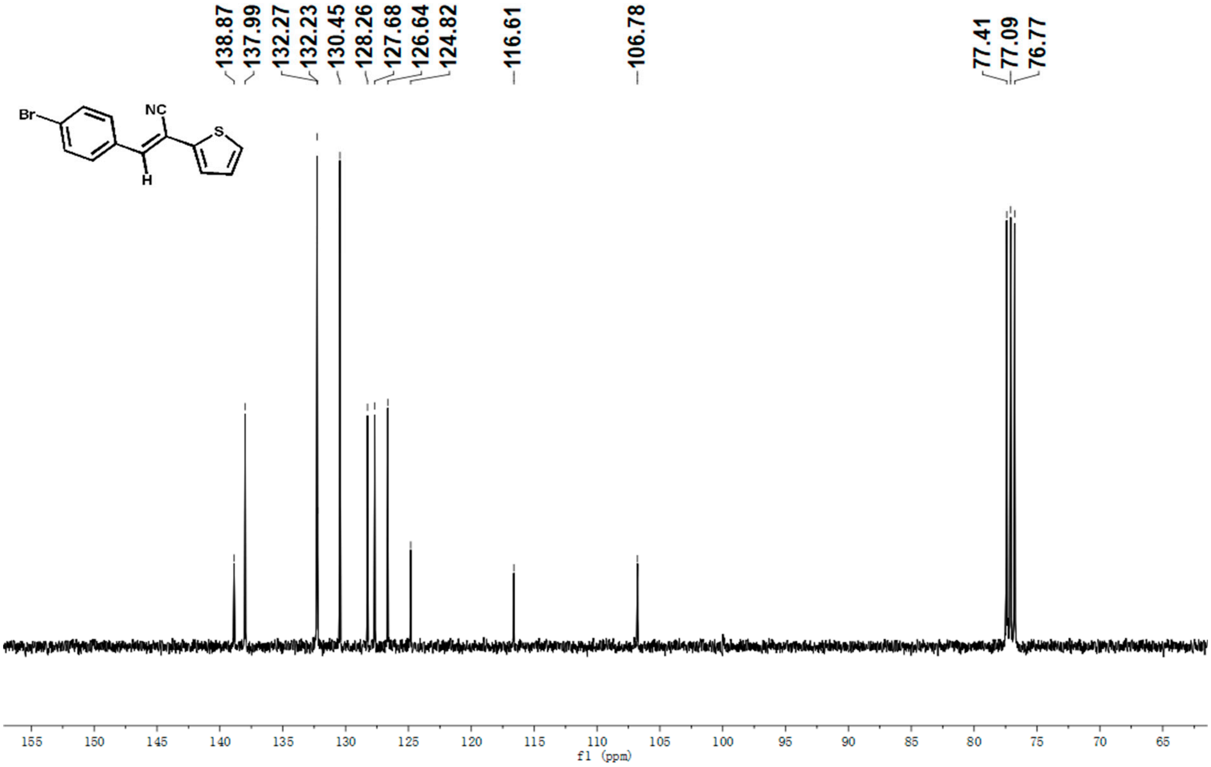

Figure S12. $^{13}C\{^{1}H$ NMR}spectrum of **CST-BP** (100 MHz, $CDCl_3$).

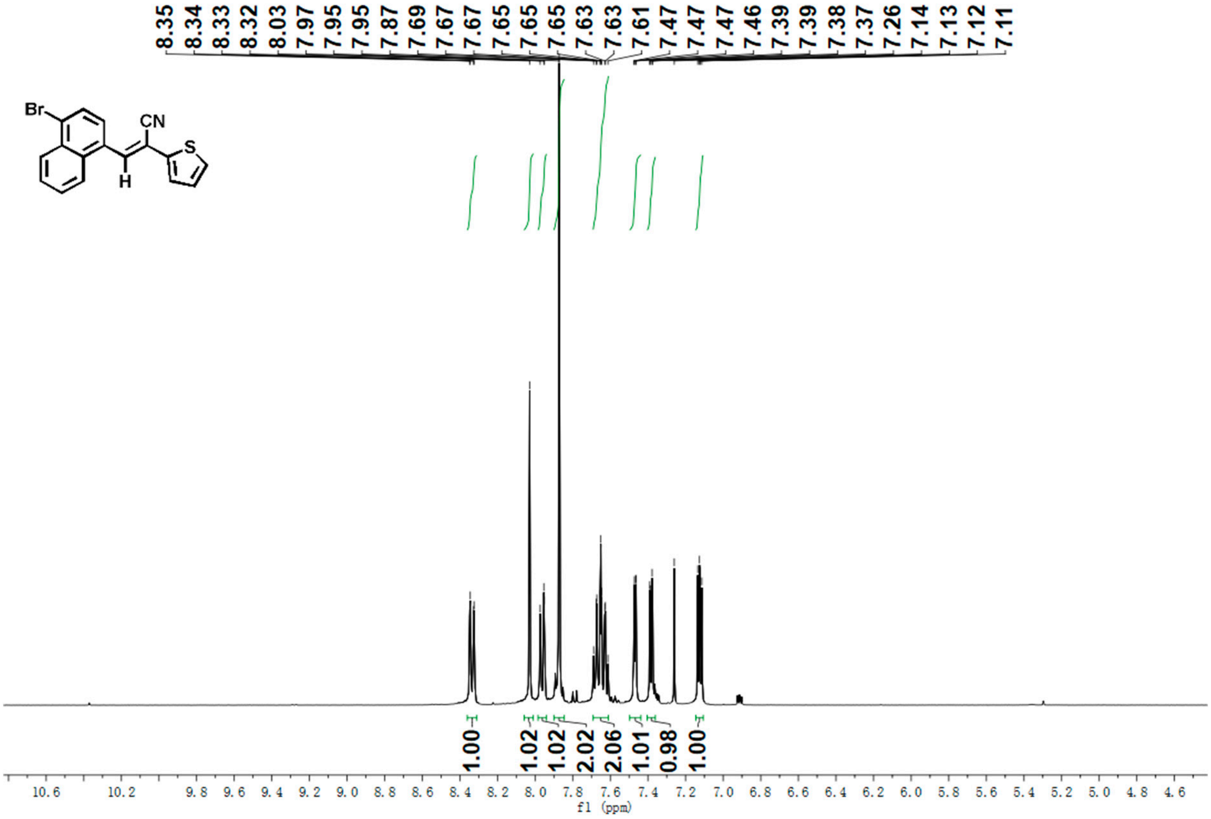

Figure S13. ^{1}H NMR spectrum of **CST-BN** (400 MHz, $CDCl_3$).

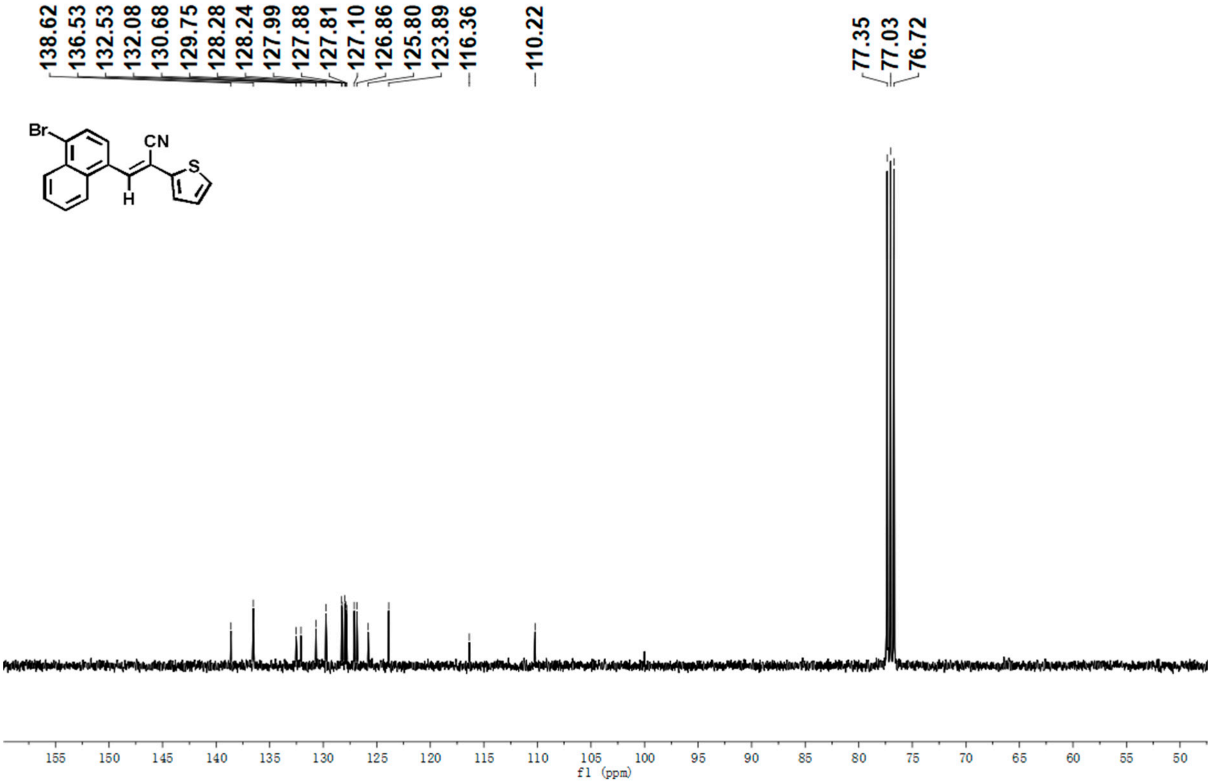

Figure S14. $^{13}C\{^{1}H$ NMR}spectrum of **CST-BN** (100 MHz, $CDCl_3$).

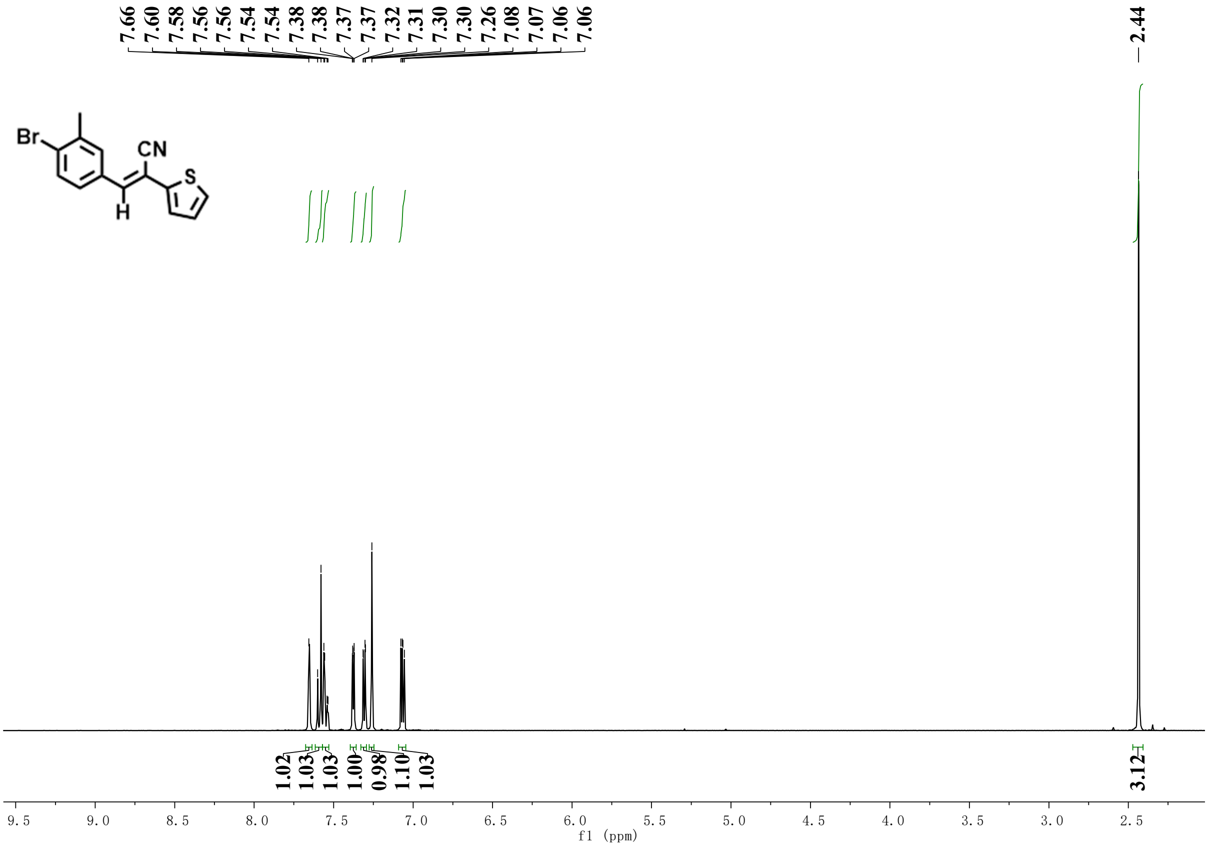

Figure S15. ^{1}H NMR spectrum of **CST-BMP** (400 MHz, $CDCl_3$).

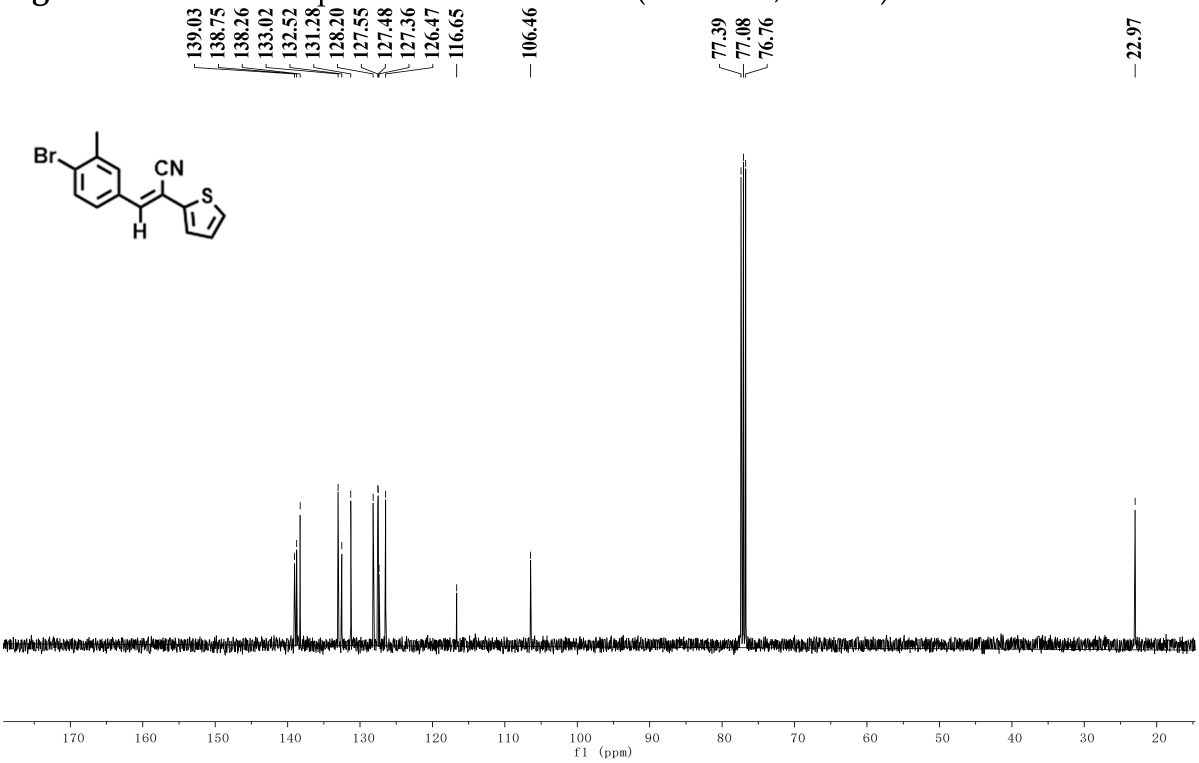

Figure S16. $^{13}C\{^{1}H$ NMR$\}$spectrum of **CST-BMP**(100 MHz, $CDCl_3$).

Table S1. Summary of the PHP performances of linear CPs.

Photocatalyst	Synthetic methods	Co-catalyst	SED	λ (nm) [a]	HER (mmol h^{-1} g^{-1})	Ref.
**CP3**	DArP	--	AA	>420	7.60	This work
P3HT	Ni-catalyzed	Pt	Na_2S/ Na_2SO_3	>400	0.005	S1
PFBT/CN	Suzuki	Pt	TEOA	>420	0.72	S2
P7-E	Sonogashira	--	TEOA	>420	6.02	S3
L-PDBT-O	Sonogashira	--	TEOA	>420	4.43	S4
Flu-SO	Suzuki	--	TEA	>420	5.04	S5
P10	Suzuki	--	TEA	>420	3.26	S6
CP4	DArP	--	AA	>420	0.17	S7
FSO-FS	Suzuki	--	TEOA	>420	3.40	S8
PyPm	Suzuki	Pt	TEOA	>300	0.37	S9
p-FuS	Suzuki	--	TEA	>420	5.88	S10
FSO-TPdT	Suzuki	Pt	TEOA	>420	7.39	S11
PEB−DBT−0.1PY	Sonogashira	--	TEOA	>420	0.54	S12

AA: ascorbic acid, SA: sodium ascorbate, TEOA: triethanolamine, and TEA: trimethylamine. [a] All light sources are 300 W Xe lamp.

References

S1 Yan, H. J.; Huang, Y.; Polymer composites of carbon nitride and poly(3-hexylthiophene) to achieve enh anced hydrogen production from water under visible light. *Chem. Commun,* **2011**, *47*, 4168-4170, doi:10.1039/c1cc10250h.

S2 Chen, J.; Dong, C. L.; Zhao, D.; Huang, Y. C.; Wang, X. X.; Samad, L.; Dang, L. N.; Shearer, M.; Shen, S. H.; Guo, L. J. Molecular design of polymer heterojunctions for efficient solar–hydrogen conversion. *Adv. Mater.* **2017**, *1606198*, doi:10.1002/adma.201606198.

S3 Zhang, X. H.; Wang, X. P.; Xiao, J.; Wang, S. Y.; Huang D. K.; Ding, X.; Xiang, Y. G.; Chen, H.. Synth esis of 1,4-diethynylbenzene-based conjugated polymer photocatalysts and their enhanced visible/near-i nfrared-light-driven hydrogen production activity *J. Catal,* **2017**, *350,* 64-71, doi:10.1016/j.jcat.2017.02.026.

S4 Wang, X. P.; Chen, B.; Dong, W. B.; Zhang, X. H.; Li, Z. B.; Xiang, Y. G.; Chen H.. Hydrophilicity-con trolled conjugated microporous polymers for enhanced visible-light-driven photocatalytic H_2 evolution. *Macromol. Rapid Commun,* **2018**, 1800494, doi:10.1002/marc.201800494.

S5 Dai, C. H.; Xu, S. D.; Liu, W.; Gong, X. Z.; Panahandeh-Fard, M.; Z. Liu, T.; Zhang, D. Q.; Xue, C.; L oh, K. P.; Liu, B.. Dibenzothiophene-S,S-dioxide-based conjugated polymers: highly efficient photocataly ts for hydrogen production from water under visible light. *Small,* **2018**, 1801839, doi:10.1002/smll.201801 839.

S6 Sachs, M.; Sprick, R. S.; Pearce, D.; Hillman, S. A. J.; Monti, A.; Guilbert, A. A. Y.; Brownbill, N. J.; Di mitrov, S.; Shi, X. Y.; Blanc, F.; Zwijnenburg, M. A.; Nelson, J.; Durrant, J. R.; Cooper, A. I.. Understan ding structure-activity relationships in linear polymer photocatalysts for hydrogen evolution. *Nat. Com mun.,* **2018**, *9,* 4968, doi:10.1038/s41467-018-07420-6

S7 Huang, W. Y.; Shen, Z. Q.; Cheng, J. Z.; Liu, L.; Yang, K.; Chen, X. R.; Wen, H. R.; Liu, S. Y.. C–H act ivation derived CPPs for photocatalytic hydrogen production excellently accelerated by DMF cosolvent. *J. Mater. Chem. A,* **2019**, *7,* 24222-24230, doi:10.1039/C9TA06444C.

S8 Lan, Z. A.; Zhang, G. G.; Chen, X.; Zhang, Y. F.; Zhang, K. A. I.; Wang, X. C.. Reducing the exciton bi nding energy of donor–acceptor-based conjugated polymers to promote charge-induced reactions. *Ange w. Chem. Int. Ed.,* **2019**, *58,* 10236-10240, doi:10.1002/anie.201904904.

S9 Wang, Z. J.; Mao, N.; Zhao, Y. B.; Yang, T. J.; Wang, F.; Jiang, J. X.. Building an electron push–pull sys tem of linear conjugated polymers for improving photocatalytic hydrogen evolution efciency. *Polym. B ull.,* **2019**, *76,* 3195-3206, doi:10.1007/s00289-018-2535-3.

S10 Bai, Y.; Woods, D. C. J.; Wilbraham, L.; Aitchison, C. M.; Zwijnenburg, M. A.; Sprick, R. S.; Cooper, A . I.. Hydrogen evolution from water using heteroatom substituted fluorene conjugated co-polymers. *J. Mater. Chem. A,* **2020**, *8,* 8700-8705, doi:10.1039/d0ta02599b.

S11 Chen, R. K.; Hu, P. W.; Xian, Y. X.; Hu, X. H.; Zhang, G. B.. Incorporation of sequence aza-substitutio n and thiophene bridge in linear conjugated polymers toward highly efficient photo-catalytic hydrogen evolution. *Macromol. Rapid Commun,* **2022**, *43,* 2100872, doi:10.1002/marc.202100872.

S12 Xiao, J.; Xiao, Z. H.; Hu, J. H.; Gao, X. F.; Asim, M.; Pan, L.; Shi, C. X.; Zhang, X. W.; Zou, J. J.. Ratio nal design of alkynyl-based linear donor−π−acceptor conjugated polymers with accelerated exciton diss ociation for photocatalysis. *Macromolecules,* **2022**, *55,* 5412-5421, doi:10.1021/acs.macromol.2c00885.
